# Transcendence and Sublime Experience in Nature: Awe and Inspiring Energy

**DOI:** 10.3389/fpsyg.2019.00509

**Published:** 2019-03-13

**Authors:** Lisbeth C. Bethelmy, José A. Corraliza

**Affiliations:** ^1^Department of Behavioral Science and Technology, Universidad Simón Bolívar, Caracas, Venezuela; ^2^Department of Social Psychology and Methodology, Universidad Autónoma de Madrid, Madrid, Spain

**Keywords:** sublime, awe, inspiring energy, transcendent emotions, nature, operational definition

## Abstract

The wilderness is one of the most widely recognized sources of transcendent emotion. Various recent studies have demonstrated nature’s power to induce intense emotions. The study at hand will generate conceptual and operational definitions of sublime emotion toward nature. Taking into consideration the recent research on feelings of awe, an instrument is devised to measure sublime emotion toward nature. The proposed scale’s reliability and validity is tested in a sample of 280 participants from the general population of Madrid. Results show that sublime emotion was defined by two conceptual components: awe, and inspiring energy, both obtained using the computer program FACTOR. After reliability and validity analysis, the Sublime Emotion toward Nature (SEN) scale included 18 items, distributed into awe (6 items, α = 0.881) and inspiring energy (12 items, α = 0.933). Awe was defined by feelings of fear, threat, vulnerability, fragility, and respect for nature, which is perceived as vast, powerful, and mysterious. Inspiring energy was defined by feelings of vitality, joy, energy, oneness, freedom, eternity, and harmony with the universe. The SEN is an adequate instrument to measure transcendent emotions provoked by direct wilderness exposure or memory thereof.

## Introduction

In research on humans’ relationship to the natural world, spirituality is key to understanding people’s emotions and the meaning of nature to them (Fredrickson and Anderson, 1999). Stokols (1990) maintains that it is a central element of environmental experience. Spirituality can be defined as “an individual’s inner experience and/or belief system, that gives meaning to existence, and subsequently allows one to transcend beyond the present context,” in Kamitsis and Francis’s (2013, p. 137) words. In recent years, spirituality research has peaked in association with research on transcendent experiences (Levin and Steele, 2005; Yaden et al., 2017) in relation to wellbeing (Yaden et al., 2017), health (Levin and Steele, 2005), and other aspects. It has, further, opened up a specific line of research on feelings of awe, which researchers have undertaken experimentally (Piff et al., 2015) as well as phenomenologically (Bonner and Friedman, 2011), building on Keltner and Haidt (2003) pioneering contribution.

Various studies have examined what elicits this sort of transcendent emotion, as well as its effects. They all emphasize that nature is a principal source for people to “experience a sense of spirituality” (Kamitsis and Francis, 2013, p 236). However, there is not a consensual definition about these spiritual and transcendent experiences to nature, being more difficult a quantitative approach (Joye and Bolderdijk, 2015). This study presents an instrument and a definition to measure spiritual responses elicited by nature, a particular transcendent emotions, that we propose to call sublime emotion toward nature.

Sublime experience has been defined as a mix of emotions – arousal, pleasure, and vitality - together with feelings of awe in nature, which is perceived as powerful, vast, and complex (Bodei, 2008/2011). According to Van Elk et al. (2016), “sublime is experienced as boundlessness that can eventually overwhelm or even destroy the observer” (p.11). Those authors recognize that sublime emotion can be evoked by the imposing natural world, and that it resembles the feeling of awe.

Edmund Burke (1757/2005), in his essay on “the sublime and beautiful” published in 1757, defined the sublime as an emotion mainly characterized by feelings of amazement and fear, and to a lesser extent admiration, respect, and reverence. Burke adds that sublime emotion means experiencing passions painful or delicious, terrible or pleasurable, depending on a person’s proximity to real danger. It is triggered by the experience of subjugation to something greater than oneself, such as nature, the divine, or the institutionalized power of kings, and is associated with awe and “a connection with terror” (p. 97). Likewise, Burke describes the sublime in terms of privation, “the void, darkness, solitude, and silence” (p. 101); vastness, unimaginable smallness, majesty, the unknown, the notion of the infinite that elicits “delightful horror” (p. 103).

According to Kant (1764/2008), sublime is an emotion that presupposes the soul’s excitability. It can manifest as “the terrifying sublime,” which is those emotions that are accompanied by a degree of horror or melancholy; “the noble,” which is related to quiet admiration; or “the splendid,” which is a sense “of beauty that extends to the plane of the sublime” (p. 32). He includes a sense of dread and respect as yet another property of the sublime.

Bodei (2008/2011) defines the sublime as an experience of simultaneous fear and pleasure. According to his analysis, nature inspires sublime emotion. First, “horrid places” (p. 19) such as “oceans, deserts, mountains, untamed jungles, and volcanoes have qualities that awaken fear, fright, and goosebumps” (p.19). Second, cosmic nature, the “planet that, floating, navigates through the void, through immense dark spaces” (p. 27), fills people with anguish, uneasiness, shock, and terror as they confront concepts they cannot rationally understand – like death, the infinite, and limitless expansion.

Johnson (2002), on the other hand, posits the sublime as one of various spiritual feelings that emerge during wilderness contact. He defines it as “the awesome power of wild nature and its indifference to human concerns” (p.29), and suggests there is a need for empirical research to test the spiritual benefits of wilderness exposure.

Recent interest in transcendent emotions has built on important historical contributions from Psychological Science. William James (1902/1986, 1890/1950) refers to spiritual identity as a sense of oneness with all things, and he connects it to a mystic experience – a sort of spiritual, religious experience that is typically ineffable, true, transitory, passive, and brought about by a perceived higher power. Nature evokes these feelings because “it seems to have a peculiar power of awakening such mystical moods” (James, 1902/1986, p. 385). James adds that mystical experiences provoked by nature are cited in works of art, including and especially Walt Whitman’s poetry (James, 1902/1986), which conveys a sense of interconnectedness and oneness between the entire universe and the personal, private sphere.

Maslow (1964), too, studied human emotions related to self-transcendence and self-realization, in terms of his famous concept of peak experience. Emotional response during a peak experience is described as a constellation of intense feelings, associated with terms like “wonder, surprise, awe, amazement, reverence, humility and surrender before the experience as before something great” (Maslow, 1999, p.89). Self-perception transcends the self, and what emerges is a sense of oneness with the broader universe. Similarly, it is associated with positive emotions like self-realization, and impulses toward honesty and goodness.

A third point of conceptual support for investigating transcendent emotions is flow experience. Csikszentmihalyi (2014) defines this as a person entering a state of absorption, full attention, and abstraction in a personally fulfilling activity that brings him or her closer to a positive state – for instance, a state of utter joy, a sense of balance and self-sufficiency in life, etc. Seligman and Csikszentmihaly (2000) link flow experience to spirituality as a Positive Psychology tool to “move individuals toward better citizenship” (p. 5).

Mystical experience, peak experience, and flow are all psychological processes related to transcendent emotions (Yaden et al., 2017). Several studies offer empirical support that nature prompts transcendent emotional reactions. Talbot and Kaplan (1986) argue that wonder and awe are among people’s emotional reactions to direct contact with wilderness, along with a feeling of oneness with nature (see also Hartig and Evans, 1993).

Williams and Harvey (2001) conducted a study to discover types of transcendent emotions produced in the forest, in the state of Victoria in southeast Australia. Among others measures, they adapted the Hood (1975) mysticism scale to measure “the transcendent nature of the experience” (p. 251) and defined transcendent emotions in the forest as intense positive mood, unity, absorption, and timelessness. Through cluster analysis, they determined that transcendent emotion is defined by feeling small and insignificant while also experiencing deep flow. Feeling small is marked by a high level of fascination and novelty, low belonging, and feelings associated with awe, wonder, and humility toward the natural world. Meanwhile deep flow is defined by feelings of belonging and relaxation in the forest.

Seamon (1984) analyzes Wordsworth poems (1770–1850) from a phenomenological perspective, concluding that spiritual emotions denote a profound sense of belonging with nature and refer to positive, pleasant feelings like joy, exaltation and the sublime feeling in particular. He mentions a heightened emotion feeling, which he describes as a sense of inner peace that suspends the individual in a deep, inner spiritual state; hard to explain rationally with words, it is best understood through lived experience.

Vining and Merrick (2012), on the other hand, propose studying *environmental epiphanies* as “highly significant and meaningful experiences that shift the fundamental self-nature relationship” (p. 506). Vining and Merrick lay out five types of environmental epiphany: aesthetic, intellectual, realization, awakening, and connectedness epiphanies. The latter is described in terms of spiritual and mystic qualities, and the experience of becoming one with nature. It even involves physical sensations (numbness or tingling) that can change a person’s perception of where s/he is.

Taking an empirical and especially qualitative approach, Cousins et al. (2009) studied people’s emotional states while contemplating wild animals. They feel transcendent emotions they describe as awe and wonder, which mix emotional responses like “epiphany and joy with mortal fear” (p. 1075) when they are in the wilderness, or while contemplating large mammals in the wild.

Kamitsis and Francis (2013) conducted a study of 190 participants, concluding that spirituality mediated the effects of nature exposure and nature connectedness on psychological wellbeing.

The literature indicates that, like Kamitsis and Francis (2013) found, transcendent emotions are positively associated with greater exposure and connectedness to nature. Of those emotions, awe is among the most extensively empirically examined to date (Joye and Bolderdijk, 2015).

Keltner and Haidt (2003) seminal study defines awe as a prototypical emotion in which a person feels disoriented, afraid, small, humble, and confused. It is prompted by stimuli perceived to be quite *vast*; to process a thing or place with such grandeur requires perceptual accommodation (Keltner and Haidt, 2003). Joye and Bolderdijk (2015) define awe as a “highly intense emotion that is triggered by vast and overwhelming phenomena, leading to increased spirituality, and to feelings of oneness with, and caring for others” (p. 2). They conclude that awe as a variable mediates the effect on mood, improving mood when someone is exposed to images of amazing, awesome natural landscapes, as opposed to depictions of nature in everyday spaces. Similar results have been reported about awe in appraisals of gigantic buildings (Joye and Dewitte, 2016). Awe has even been referred to as one thing people feel when they see planet Earth from space (Yaden et al., 2016).

Awe has been linked to other positive emotions that human beings experience (Maslow, 1964; Keltner and Haidt, 2003). Nisbet et al. (2009) believe the awe variable may relate to positive emotions, and recommend studying it in relation to wellbeing and connectedness with nature. Shiota et al. (2007) administered a survey of open-ended questions to 60 psychology students, concluding that the following events most often elicit awe: exposure to nature (27%) and exposure to art or music (20%). Those authors conclude that awe is a more intimate expression than happiness, and that it is only provoked by positive, not negative, events that inspire horror and fear (storms and natural disasters, among other phenomena).

Awe has been defined as a positive – as well as complex – emotion, and several recent studies have examined its effects. It has a demonstrated impact on perception of one’s body (Van Elk et al., 2016), and of time (Rudd et al., 2012). Additionally, Piff et al. (2015) research has identified two effects of awe: diminishment of the individual self; and increased prosocial behavior. Other studies have shown that awe has effects on the genesis of spiritual intentions (Van Cappellen et al., 2013). Hinds and Sparks (2008, 2011) investigations associate it with Eudaimonia, a type of psychological wellbeing associated with feelings of inner peace, happiness, vitality, and satisfaction with life. Awe, considered a sort of affective state, loaded onto the Eudaimonia factor along with other affective states – emotional connection, contemplation, serenity, vitality, sense of freedom, empathy, and freshness. Awe was related to being (or remembering being) in the jungle, mountains, and large valleys; and to a lesser extent being in the forest, river, ocean, or at the beach.

Altogether, these studies highlight some of the most important psychological benefits of awe. Yet as Bonner and Friedman (2011) point out, “awe lacks a consensual and precise meaning” (p. 222). It might be part of a broader concept of spiritual, transcendent emotions (Ashley, 2007), the sublime experience toward nature, as the present study proposes.

For better clarification, as Table 1 clearly conveys, sublime emotion as described by Burke (1757/2005) and Bodei (2008/2011) has a similar meaning to Maslow (1964) peak experience, and sense of awe as defined by Kant (1764/2008), Keltner and Haidt (2003), Haidt (2003), Shiota et al. (2007), and Cousins et al. (2009),. The definitions agree that awe, a property proposed as part of the conception of sublime emotion, is accompanied by an experience that overwhelms the self and human understanding, triggering a devotional reaction. Maslow (1964) goes further, arguing that these experiences and emotions relate to other psychological variables, like intrinsic, universal values that everyone and every culture have for wilderness experience.

**Table 1:** Summary of Basic Content Referenced about Sublime and Transcendent Emotion.

Term	Definition	Author (s)
Sublime emotion	Emotion characterized by astonishment, awe, fear, respect and admiration. Feeling a mix of terror and pleasure that becomes a “delightful horror” if no real danger is perceived. Stimuli that trigger it include natural and non-natural places with particular features and properties, qualities of human frailty, the infinite, eternity, and certain works of art. This is distinct from “beauty.”	Burke (1757/2005)
	The sort of feeling that stirs the soul, whether due to surprise, terror, reverence, magnificence, trembling, or respect. Kant added certain personal characteristics to the sublime stimuli earlier proposed by Burke.	Kant (1764/2008)
Sublime emotion toward nature	Experiencing fear and pleasure in a wilderness perceived as powerful. In a mix of awe, humility, self-transcendence, joy, sadness, enthusiasm, and connection with the whole universe. This is produced by “horrid” landscapes as well as superhuman dimensions like eternity, infinity, and the ineffable.	Bodei (2008/2011)
Self-transcendence	Peak experience. A larger-than-life experience of self-realization characterized by a mix of spiritual emotions like enlightenment, awe, reverence, humility, happiness, wonder, and connection to the universe, among others.	Maslow (1964)
	Flow experience. Achieving harmony and balance in consciousness by engaging in creativity and focused attention on a process of authentic personal growth. This entails deep enjoyment of life and attainment of happiness.	Csikszentmihalyi, 2014
Feeling of awe	An emotional experience characterized by fear, vulnerability, humility, disorientation, inspiration, and renewal; and in contrast, sensing a higher power, beauty, and justice, among other things.	Keltner and Haidt (2003) Shiota et al. (2007)
	A mix of emotional states – epiphany, joy, mortal fear, vitality, humility, vulnerability, respect, and sensing a connection with all of nature – elicited by nature’s mystery, power, savagery, and unpredictability.	Cousins et al. (2009)

The aforementioned studies share an interest in transcendent and spiritual emotions, highlighting the importance of one in particular within the empirical literature: awe. However, many authors have suggested (Bonner and Friedman, 2011, among others) there is a need to precisely define the content of this complex emotion. Moreover, we need to institute measures of awe that can be compared. For example, Joye and Bolderdijk (2015) maintain that:

We have probed for awe and awe related emotions by using only one item. This underscores the need to develop a validated scale –or any other measurement instrument- that is capable of accurately capturing the occurrence of this complex emotional state, and of associated emotions and states (Joye and Bolderdijk, 2015, p. 7).

This study endeavors to propose an instrument to measure transcendent emotions, including precisely the content referred to until now as part of awe.

This study aims to help establish transcendent and spiritual contents related to nature, and proposes the sublime as a unifying feeling that encompasses awe and positive and pleasurable emotions within a single construct.

To date, there is no empirical assessment of wilderness experience that includes emotional responses of awe, positive and pleasurable emotions, and epiphany within the same construct. Based on the Bethelmy (2012) conceptual definition, the sublime emotion toward nature is defined as a transcendent, spiritual emotion involving epiphany and a mix of heightened fear (awe) and pleasurable emotions, like wellbeing and others, provoked by a stimulus of impressive and frightful qualities in the experience with nature. The objective, therefore, is to create an operational definition of sublime emotion toward nature and design a reliable and valid instrument to measure it.

Instrument creation followed Kerlinger and Lee (2000) and Abad et al. (2011) recommendations, which maintain that instruments, in addition to being reliable, should have good indicators of discriminant and predictive validity. We expect that this operational measure of sublime emotion toward nature will yield good reliability indexes, one indication of internal consistency. Furthermore, the contents (items) of the sublime emotion scale should differ from other measures of similar emotional content, like love and care for nature (Perkins, 2010), connectedness with nature (Mayer and Frantz, 2004), and transcendent emotion (Williams and Harvey, 2001) – though it has certain elements in common with those variables – if we are to conclude that the scale has content and construct validity (Kerlinger and Lee, 2000; Abad et al., 2011).

Love and care for nature (Perkins, 2010), connectedness with nature (Mayer and Frantz, 2004) and environmental intention (Amérigo et al. (2012) are selected because these measures content items about the emotions to nature and affinity and connectedness to nature related to environmental intention and behavior. For example, love and care for nature 2010) was significantly related with connected with nature and environmental behavior (see Perkins, 2010).

Another characteristic of a good operative definition is the explanatory power as an indicator of predictive validity (Kerlinger and Lee, 2000; Abad et al., 2011). Previous results indicate that emotions toward nature have a predictive power over environmental behaviors (Grob, 1995) and even a mediating role (Corraliza and Bethelmy, 2011). Therefore, the relation of the new measurement of sublime emotion toward nature with other variables of the environmental intention and behavior is explored as an indicator of predictive validity.

## Materials and Methods

### Participants and Procedure

The sample was comprised of 280 members of the general population of Madrid, of whom 55.2% were women and 44.8% men. 225 lived in an urban area (80.4%) and 55 in a rural area (19.6%). Their average age was 41.1 (*SD* = 14.9), and their minimum and maximum ages were 17 and 77 years old, respectively.

Self-report surveys were administered by interviewers’ in-person so they could provide direct support. Non-probabilistic convenience sampling was applied. The survey took an average of 15 min to administer.

### Instruments

#### Sublime Emotion Toward Nature (SEN) Scale

Initially, an instrument with 62 items was created based on the conceptual emotional categories in the meaning of nature cited in Bethelmy (2012) qualitative study, which included items referring to feelings of wellbeing, transcendent emotions, and sense of connectedness with nature. Following an earlier study in a sample of 76 participants in the city of Madrid (see Bethelmy, 2012), after an analyses of reliability (α = 0.92) and selecting the items with a mean score higher than 4.2, standard deviation higher than 1 and item-total correlations more than *r* = 0.45 (Nunnally, 1978), the scale was reduced to 26 items, which comprise the instrument administered in the present study. Participants’ answers were recorded on a 5-point Likert-type scale from 1 (strongly disagree) to 5 (strongly agree). A new reduction of SEN items was done in the present research resulting in 18 items, eliminating those that contribute least to the internal consistency and validity, according to methodological and psychometric recommendations (Nunnally, 1978; Lorenzo-Seva and Ferrando, 2006; Abad et al., 2011).

#### Love and Care for Nature (LCN) Scale.

A Spanish version of Perkins (2010) original was created; its 15 items comprise a single factor that explains 71.18% of variance. The original scale in English has a reliability of α = 0.97. Its items were translated into Spanish by three translators and specialists in this field. The scale measures affective and emotional aspects like feelings of connectedness with nature, interest, fear, and care toward nature (Perkins, 2010). Responses are given on a 7-point Likert-type scale from 1 (*strongly disagree*) to 7 (*strongly agree*).

#### Environmental Attitudes

The scale devised by Amérigo et al. (2012) was utilized. It is comprised of four factors (20 items in total) that together explain 52.3% of total variance: anthropocentrism (5 items, α = 0.705), environmental apathy (5 items, α = 0.902), emotional affinity toward nature (5 items, α = 0.845), and connectedness with nature (5 items, α = 0.850). The Spanish version of the last dimension was previously used by Corraliza and Bethelmy (2011, α = 0.830). The instrument’s 5-point Likert-type response scale ranges from 1 (*strongly disagree*) to 5 (*strongly agree*).

#### Environmental Intentions

We used the Spanish version of Stern et al. (1999) scale, translated for the purposes of this study. Its two items measure people’s willingness to sacrifice to protect the environment. It has a 5-point Likert-type response scale ranging from 1 (*strongly disagree*) to 5 (*strongly agree*).

#### Environmental Behaviors

We used a translation of scales designed by Stern et al. (1995) and Stern et al. (1999), also utilized by Perkins (2010). A Spanish version was designed for use in the present study. The questionnaire’s five items measure the incidence of environmentalist action taking on a 4-point Likert-type response scale from 1 (*never*) to 4 (*always*).

### Statistical Analysis

For the sublime emotion toward nature measure, we conducted exploratory factor analysis (EFA); eliminating items that contribute least to internal consistency and validity, considering the value of each factor’s item-total correlations, corrected item-total correlations, factor loadings (Nunnally, 1978; Abad et al., 2011), and conceptual dimension according to the definition criteria (Bethelmy, 2012). The FACTOR program (Lorenzo-Seva and Ferrando, 2006) was employed during EFA of sublime emotion toward nature; it is one of the most highly recommended for factor analysis of psychosocial variables. The FACTOR program considers the polychoric correlation matrix, the ordinal measurement scale of the items and values of asymmetry and kurtosis that are not in the recommended levels. In addition, the FACTOR program calculates goodness-of-fit indicators and coefficients of the residuals of the resulting factor distribution (Lorenzo-Seva and Ferrando, 2006).

We calculated Cronbach’s reliability coefficient to gauge internal consistency, tested correlations to determine if there was evidence of convergent and criterion (concurrent) validity in relation to other variables.

To examine the relationships between variables, we calculated Pearson’s correlation coefficients and potential multicollinearity effects between variables in SPSS, version 19.0. The method of multiple and simple linear regression were applied to determine the effect of sublime emotion toward nature on environmental behaviors.

## Results

### Psychometric Indicators on the Sublime Emotion Toward Nature (SEN) Scale

All the variables we measured showed skewness between 0.009 and −0.6 (minimum and maximum), and kurtosis between −0.009 and −0.65 (minimum and maximum). Results in terms of Bartlett’s statistic (4297.7; df = 276 and *p* < 0.0001) and Kaiser-Meyer-Olkin (KMO = 0.95) convey that the sample meets requirements for EFA to proceed. Regarding the others variables of the study, the Spanish version of love and care for nature obtained an adequate reliability index (α = 0.923) and just one factor. Furthermore, results of exploratory factor analysis determined that the scale’s awe item should be excluded (factor loading < 0.25), so it was eliminated from the Spanish version. It does not share content with the scale’s other items, suggesting it belongs to a different variable related to fear and respect toward nature. The environmental intention reliability is adequate, and its internal consistency α = 0.72. The environmental behaviors scale is sufficiently reliable, with an internal consistency of α = 0.81. According to principal components analysis, the one-factor solution accounts for 57.6% of explained variance (eigenvalue = 2.8), and the scale’s five constituent items have factor loadings over 0.60 (ranging from 0.79 to 0.62).

Results of EFA of the SEN scale found two factors. In the first EFA, CFI was found to be under 0.85, and two items had very high residuals (item 13: *me siento insignificante ante la majestuosidad de la naturaleza* [I feel insignificant next to nature’s majesty]; and item 20: *me siento insignificante ante lo imponente de la naturaleza* [I feel insignificant compared to nature’s awesome power]), suggesting their content may have been very similar (standardized residual of 3.68). After eliminating item 20, CFI rose to 0.89, but a high residual remained between item 17 (*Me siento con una vitalidad única cuando estoy en la naturaleza* [In the wilderness, I feel a singular vitality]) and item 19 (*Me siento con mucha energía cuando estoy en la naturaleza* [I feel very energized when I’m in nature]) (standardized residual of 3.23). By eliminating item 19, we found that the scale’s goodness of fit indexes were suitable: CFI = 0.90, Chi-squared = 624.6, degrees of freedom = 229, *p* > 0.001, RMR = 0.0467, GFI = 0.99, AGFI = 0.99.

Using the method of oblimin rotation, items 3, 6, 10, 12, 13, 14, 16, 18, and 23 loaded onto Factor 1, accounting for 44.80% of variance (eigenvalue = 10.8). Meanwhile, items 1, 2, 4, 5, 7, 8, 9, 11, 15, 17, 21, 22, 24, 25, and 26 loaded on Factor 2, explaining 12.2% of variance (eigenvalue = 2.93). Factor loadings of items onto their respective factors ranged from 0.45 to 0.89. Factor loadings of items onto each factor appear in Table 2.

**Table 2:** Clustering for Exploratory Factor Analysis with Oblimin Rotation, of the Sublime Emotion toward Nature (SEN) Scale.

	Factors	
SEN items	I (α = 0.89)	II (α = 0.94)
23. *Siento miedo de mi vulnerabilidad cuando estoy en la naturaleza* [In the wilderness, I fear my own vulnerability]	0.964	
18. *Siento temor ante el misterio que encierra la naturaleza* [I am fearful of the mystery that nature holds]	0.914	
12. *Siento temor ante lo impredecible de la naturaleza* [I feel awe of nature’s unpredictability]	0.890	
16. *Estar en la naturaleza me hace sentir la fragilidad de la vida* [Being in nature makes me aware of life’s fragility]	0.714	
10. *Siento temor reverencial cuando estoy en la naturaleza* [In the wilderness, I feel awe and reverence]	0.663	
13. *Me siento insignificante ante la majestuosidad de la naturaleza* [I feel insignificant next to nature’s majesty]	0.496	
6. *Estar en la naturaleza me hace sentir la vulnerabilidad del mundo* [Being in the wilderness makes me realize how vulnerable the world is]	0.491	
3. *Cuando estoy en la naturaleza siento que los humanos somos tan vulnerables como los otros seres vivos que compartimos la Tierra* [When I am in the wilderness, I sense that humans are as fragile as any other living being on Earth]	0.428	
14. *Cuando estoy en la naturaleza siento la presencia de una fuerza superior y sobrenatural* [In the wilderness, I feel the presence of a higher, supernatural power]	0.426	
5. *Estar en la naturaleza es una de las cosas que me hacen sentir felicidad auténtica* [Being in the wilderness is one of the things that make me feel truly happy]		0.870
9. *Tengo un profundo sentimiento de conexión con la naturaleza* [I feel deeply connected to nature]		0.841
7. *Tengo un profundo sentimiento de pertenencia con la naturaleza* [In nature I feel a deep sense of belonging]		0.768
24. *Estar en la naturaleza me inspira en la vida* [Being in nature brings inspiration to my life]		0.764
15. *Siento profunda armonía con el universo cuando estoy en la naturaleza* [In the wilderness, I sense a profound harmony with the universe]		0.745
2. *Me siento más vivo que nunca cuando estoy en la naturaleza* [I feel more alive than ever in nature]		0.744
17. *Me siento con una vitalidad única cuando estoy en la naturaleza* [In the wilderness, I feel a singular vitality]		0.734
21. *La grandiosidad de la naturaleza me hace sentir libre* [The magnificence of nature makes me feel free]		0.720
8. *No tengo palabras para decir todo lo que siento cuando estoy en la naturaleza o veo documentales acerca de ella o me doy cuenta de su presencia* [Words cannot describe everything I feel when I am in the wilderness, I watch a nature documentary, or I become aware of nature’s presence]		0.699
26. *Estar en la naturaleza me hace sentir conectado profundamente con todos los seres vivos* [Being in nature makes me feel deeply connected to all living beings]		0.694
4. *Siento el enorme poder de la naturaleza* [I sense nature’s enormous power]		0.597
1. *A menudo siento que lo que tengo ante mí cuando estoy en la naturaleza, me deja sin palabras* [Often what is before me in the wilderness leaves me speechless]		0.581
11. *Siento que soy parte del universo cuando estoy en la naturaleza* [In the wilderness, I feel like part of the universe]		0.569
25. *Cuando estoy en la naturaleza siento la armonía de la vida y la muerte* [In the wilderness, I feel that life and death are in harmony]		0.466
22. *Tengo una sensación de eternidad cuando estoy en la naturaleza o pienso sobre ella* [I feel a sense of eternity when I am in the wilderness, or think about it]		0.456

*I: Awe; II: Inspiring energy. Weighted factor > 0.40.*

By means of a more thorough analysis, we eliminated the items that contribute least to internal consistency, to each factor’s item-total correlations and corrected item-total correlations, to factor loadings, and to *belonging* with the dimension’s theory and content. Also, item selection criteria included mean under 4, and SD under 1.

This led us to eliminate items 3 and 14 from Factor 1 for both lower item-total correlation and lower factor loading. Item 6 was eliminated for lower factor loading, loading on both factors, and because its item-total correlation was low compared to other items (item-total *r* = 0.61; α = 0.881) (See Table 3).

**Table 3:** Criteria to Eliminate Items from Factor 1 of the SEN: Descriptive Statistics, Reliability, and Homogeneity.

Factor I (α = 0.890)	*M*	*SD*	*r*^a^	α*^b^*	Factor loading
s3	3.7	1.2	0.56	0.884	0.43
s6	3.4	1.2	0.61	0.881	0.44
s10	2.6	1.2	0.63	0.879	0.66
s12	2.9	1.3	0.69	0.874	0.89
s13	3.6	1.2	0.61	0.881	0.50
s14	2.9	1.4	0.53	0.888	0.43
s16	3.1	1.2	0.75	0.869	0.71
s18	2.6	1.3	0.70	0.873	0.91
s23	2.4	1.3	0.74	0.870	0.96

*Factor loading > 0.40. s = sublime. ^a^Item-total correlation. ^b^Item-total correlation if item is eliminated.*

Likewise, items 1, 2, and 4 were eliminated from Factor II. Item 8 which showed higher item-total correlation and internal consistency, repeats content from item 1. Item 4’s content is ambiguous and more closely aligned with Factor 1 (contents of awe). Item 2 was eliminated because its content is repeated in item 17, its corrected item-total correlation (*r* = 0.67) is lower than item 17’s (*r* = 0.71), its average score is over 4, and its standard deviation is under 1 (see Table 4). Items 22 and 25 loaded only in Factor II, both with an important contribution to the meaning of this Factor.

**Table 4:** Criteria to Eliminate Items from Factor II of the SEN: Descriptive Statistics, Reliability, and Homogeneity.

Factor II (α = 0.941)	*M*	*SD*	*r*^a^	α^b^	Factor loading
s2	4.1	0.9	0.67	0.938	0.74
s5	3.9	1.0	0.70	0.937	0.87
s7	3.6	1.2	0.71	0.936	0.77
s8	3.6	1.2	0.72	0.936	0.69
s9	3.6	1.2	0.80	0.934	0.84
s15	3.5	1.2	0.78	0.935	0.75
s17	3.8	1.1	0.71	0.937	0.73
s21	3.9	1.9	0.70	0.937	0.72
s24	3.7	1.2	0.75	0.935	0.76
s26	3.5	1.2	0.76	0.935	0.69
s1	3.9	1.1	0.62	0.939	0.58
s4	3.9	1.0	0.64	0.938	0.59
s11	3.5	1.2	0.67	0.938	0.57
s22	3.1	1.3	0.61	0.940	0.46
s25	3.1	1.3	0.62	0.939	0.47

*Factor loading > 0.40. s = sublime. ^a^ Item-total correlation. ^b^ Item-total correlation if item is eliminated.*

The refined SEN scale has a total of 18 items distributed across two factors. Items 10, 12, 13, 16, 18, and 23 loaded onto Factor 1, and its internal consistency is α = 0.881. Meanwhile Factor II includes items 5, 7, 8, 9, 11, 15, 17, 21, 22, 24, 25, and 26, with an internal consistency of α = 0.933. Below we define each factor’s contents as a function of the items belonging to it.

Factor I is defined by awe, that is, contents of vulnerability, fear of the unpredictable, of mystery, and life’s fragility and insignificance. Its meaning relates to fear of the natural world, which is perceived as unpredictable, powerful, and majestic. Feelings of humility are also present, along with voluntary submission to and respect for nature, onto which subjugating qualities are projected, along with a higher power and mystery.

Factor II has been defined as inspiring energy, characterized by joy, freedom, inspiration, deep connection with living beings and with nature, harmony with the universe, vitality, eternity, a sense of belonging with nature, harmony between life and death, speechlessness about feelings of astonishment, epiphany, intense emotional arousal about things that are transcendent or exalted. Similarly, it involves a state of vigor and inspiring energy toward life. These aspects altogether – harmony and connection on the one hand; energy, freedom, and inspiring vitality on the other – can be interpreted as a state of emotional euphoria related to wellbeing, happiness, and fascination toward the transcendence of nature and higher powers in the universe.

In view of the above, sublime emotion’s operational definition is 2-fold: it awakens feelings of liberation, inspiration, and being overcome by a sense of harmony and connection with the universe, living beings, and nature; and simultaneous feelings of awe and respect for nature, including living beings, the universe, and the existential phenomena of life and death (refer to Table 2 for the definitive, complete SEN scale).

Last, we analyzed two factors, awe and inspiring energy. The criterion that each factor has at least four to seven items was met (Abad et al., 2011). Pearson’s bivariate correlation between the two factors is *r* = 0.52 (*p* < 0.01), suggesting a moderate, significant relationship between the two.

This final version of SEN (18 items) is used for calculating the analyses of descriptive, comparative and correlational data between variables. Analysis of awe and inspiring energy results follow.

### Analyses of Descriptive, Comparative, and Correlational Data Between Variables

Respondents’ awe scores were moderate (*M* = 2.86, *SD* = 1), whereas inspiring energy scores tended to be moderate-high (*M* = 3.55, *SD* = 0.89) considering the scale range is 1–5. For minimum and maximum total scores values on each variable, see Table 5. The data tended toward normal distribution, given that the absolute value of skewness was less than 2, and kurtosis under 4 (Abad et al., 2011).

**Table 5:** Descriptive of the variables (Total scores).

Variables	*M (SD)*	Minimum	Maximum	Skewness	Kurtosis
Awe	17.2 (6.0)	6	30	0.3	−0.65
Inspiring energy	42.6 (10.7)	12	60	−0.3	−0.49
Love and care for nature	74.3 (17.4)	16	98	−0.7	−0.24
Anthropocentrism	11.4 (4.6)	5	36	0.94	1.93
Environmental apathy	8.9 (4.1)	5	24	1.3	1.50
Emotional affinity toward nature	19.6 (4.4)	6	26	−0.7	−0.09
Connectedness with nature	14.6 (3.8)	4	20	−0.4	−0.39
Environmental intention	7.4 (1.8)	2	10	−0.60	0.28
Environmental behaviors	12.4 (3.8)	5	25	0.06	−0.22

Scores on love and care for nature were high in this sample (*M* = 5.3, *SD* = 1.24), considering the scale range is 1–7. Meanwhile, anthropocentrism (*M* = 2.28, *SD* = 0.92, scale range 1–5) fell in the moderate-low range. Likewise, environmental apathy scores were very low in this sample, nearly hitting the minimum possible score (*M* = 1.77, *SD* = 0.82, scale range 1–5). Emotional affinity toward nature and connectedness with nature (*M* = 3.9, *SD* = 0.87, *M* = 3.65, *SD* = 0.9, respectively) got moderate-high scores (scale range 1–5). Environmental intention also registered high scores (*M* = 3.69, *SD* = 0.89, scale range 1–5) and environmental behaviors score fell in moderate range (*M* = 2.48, *SD* = 0.75, scale range 1–4).

### Convergent Validity

First of all, we examined correlational data to determine if there was evidence of convergent validity between the SEN scale’s two factors and similar variables, like love and care for nature, connectedness with nature, and emotional affinity toward nature (See Table 6).

**Table 6:** Correlations among the Study’s Variables (Bivariate Pearson Correlation).

	1	2	3	4	5	6	7	8
1. Awe								
2. Inspiring energy	0.47^∗∗^							
3. Love and care for nature	0.34^∗∗^	0.85^∗∗^						
4. Anthropocentrism	0.16^∗^	0.04	0.01					
5. Environmental apathy	0.14^∗^	−0.18^∗^	−0.27^∗^	0.37^∗∗^				
6. Emotional affinity toward nature	0.11	0.56^∗∗^	0.64^∗∗^	−0.01	0.30^∗∗^			
7. Connectedness with nature	0.23^∗∗^	0.57^∗∗^	0.63^∗∗^	0.05	−0.20^∗∗^	0.59^∗∗^		
8. Environmental intention	0.05	0.23^∗∗^	0.34^∗∗^	−0.12^∗^	−0.32^∗∗^	0.35^∗∗^	0.39^∗∗^	
9. Environmental behaviors	0.17^∗^	0.36^∗∗^	0.48^∗∗^	−0.09	−0.24^∗∗^	0.39^∗∗^	0.41^∗∗^	0.48^∗∗^

*^∗^p < 0.05. ^∗∗^p < 0.001.*

Awe showed significant correlations, *r* = 0.23 (*p* < 0.001) and *r* = 0.34 (*p* < 0.001), with connectedness with nature and love and care for nature, respectively. That was sufficient evidence for its convergent and discriminant validity since the contents, while correlated, belong to different constructs. The correlation between awe and emotional affinity toward nature was not significant (*r* = 0.11, *p* > 0.05).

On that note, inspiring energy had a high correlation with love and care for nature (*r* = 0.85, *p* < 0.001), and moderate, significant correlations with connectedness with nature (*r* = 0.57, *p* < 0.001) and emotional affinity toward nature (*r* = 0.56, *p* < 0.001). That is ample evidence of convergent and discriminant validity; we expected these contents, which refer to feelings during wilderness contact, would be inter-related and complementary even though they are measured as distinct constructs. Please bear in mind that in the Spanish adaptation of the Love and Care for Nature scale created for the purposes of this study, the item tapping awe and admiration toward nature did not yield acceptable values and was eliminated. As a result, that measure concentrates on content like interconnectedness, wellbeing, and care for nature (see Perkins, 2010).

A novel finding was awe’s significant, positive correlation with anthropocentrism (*r* = 0.16, *p* < 0.05) and environmental apathy (*r* = 0.14, *p* < 0.05), though the coefficients were low.

Awe displayed a moderate-low yet significant correlation with environmental behaviors (*r* = 0.17, *p* < 0.001), presenting significant albeit low-level evidence of criterion validity. Correlations between inspiring energy and environmental intentions (*r* = 0.23, *p* < 0.001) as well as environmental behaviors (*r* = 0.36, *p* < 0.001) were moderate and significant, thus providing sufficient evidence of criterion validity.

### Predictive Power of Sublime Emotion Toward Nature

Coefficients of determination (partial multiple regression) were calculated, and a scatter plot created to test criterion validity (Abad et al., 2011). Awe explained a significant, but small portion of variance in environmental behaviors (*R*^2^ = 0.030, *F* = 8.5, *p* < 0.05), and yielded a partial regression coefficient of β = 0.173 (*p* < 0.05). Again, inspiring energy showed sufficient evidence of criterion validity with respect to environmental behaviors, explaining 12.7% of variance (*R*^2^ = 0.13, *F* = 40.3, *p* < 0.001), and yielding a partial regression coefficient of β = 0.36 (*p* < 0.001). Considering these results, predicting environmental variables should be undertaken with caution.

The ability of each factor of sublime emotion toward nature – awe and inspiring energy – to predict environmental behaviors yielded the following results (multiple regressions).

Awe and inspiring energy predict environmental behaviors both directly and significantly (β = 0.17, *p* < 0.01, β = 0.36, *p* < 0.001, respectively) (see Table 7). However, when the effect of inspiring energy intervenes, awe no longer appears to predict environmental behaviors (see Table 7).

**Table 7:** Simple Linear Regression and Multiple Regression of Sublime Emotion Predicting Environmental Behaviors.

Variables	*B*	*SE B*	β	*R*^2^	*F*
**Simple linear regression**					
Inspiring energy	0.12	0.02	0.36^∗∗^	0.13	40.3^∗∗^
Awe	0.11	0.04	0.17^∗∗^	0.03	8.56^∗∗^
**Multiple regression**					
Inspiring energy^a^	0.12	0.02	0.35^∗∗^	0.12	20.1^∗∗^
Awe^b^	0.00	0.04	0.00		

*^a^ Effect of inspiring energy in multiple regression. ^b^ Effect of awe controlled for in multiple regression. Durbin-Watson = 1.97. ^∗∗^p < 0.001.*

## Discussion

The present research objectives were: to create a valid, reliable instrument with which to measure sublime emotion toward nature, one that captures the definition of the sublime as a mix of several emotions; and to differentiate sublime emotion toward nature from other variables – such as love and care for nature, environmental attitudes, and environmental behaviors. This study produced a valid, reliable way to operationally define sublime emotion toward nature.

### On the Operational Definition of Sublime Emotion Toward Nature

Sublime emotion toward nature is a composite of feelings of awe and inspiring energy, which describe different affective experiences. Awe is defined as a mix of fear and respect toward nature, which is experienced as so much larger than oneself. Meanwhile, inspiring energy is defined as the feeling that awakens a sense of vitality, happiness, freedom, and unity between the self and the natural world (and by extension the individual’s surroundings). Both are related to the two basic components of transcendent emotion, which according to Yaden et al. (2017), are: (a) annihilational (reduction of self-boundaries and self-salience) and (b) relational, “sense of connectedness, even to the point of oneness…with other people and aspects of one’s environment or surrounding context” (p. 145).

Sublime emotion toward nature is also discernably different from awe alone, depending on the trigger stimulus and the resulting experience. The present research results show that a sense of awe is part of sublime emotion; but sublime emotion includes additional content.

Inherent to awe are feelings of vulnerability, insignificance, and fear of nature’s qualities, such as magnificence, power, mystery, unpredictability, and the fragility of life. For example, item 18 (*Siento temor ante el misterio que encierra la naturaleza* [I am fearful of the mystery that nature holds]), captures these contents. This appears, too, in Keltner and Haidt (2003) definition, which other researchers in this field have followed. Inspiring energy, meanwhile, includes contents of happiness, unity, and belonging with nature, the universe, and other living beings; a sense of inspiration, freedom, vitality, eternity, ineffability, and harmony between the polar opposites of life and death. Item 8 on the SEN scale (*No tengo palabras para decir todo lo que siento cuando estoy en la naturaleza o veo documentales acerca de ella o me doy cuenta de su presencia* [Words cannot describe everything I feel when I am in the wilderness, I watch a nature documentary, or I become aware of nature’s presence]) captures the ineffability described by James (1902/1986) and Williams and Harvey (2001), which is one of the unique qualities of spiritual reactions to nature. Furthermore, “ineffability” is related to vitality and renewed energy to make a positive change (Williams and Harvey, 2001).

The awe factor we observed is consistent with the categories Ashley (2007) reported in his qualitative study. The inspiring energy factor, meanwhile, is empirically supported by earlier findings about the categories of spiritual inspiration in nature, connectedness, and a sense of belonging with nature (Fredrickson and Anderson, 1999; Schroeder, 2007; Vining et al., 2008).

Meanwhile, factors which this study found to define sublime emotion toward nature would overlap in meaning with self-transcendent feelings in nature. Inspiring energy encompasses happiness and transcendent joy, which are also part of the definition of awe. By the same token, Robbins (2006) found that the sense of pure happiness – joy – relates to having experienced fear and respect before that sheer happiness. With that in mind, we recommend further examining the conceptual link between the two elements of sublime emotion, whether or not they effectively constitute two emotional elements with distinct psychological features and functions.

In light of the above, the present study lays out empirical evidence of a reciprocal relationship between ambivalent feelings with spiritual qualities, both triggered by wilderness encounters.

Sublime emotion toward nature, thusly defined as a mix of awe and inspiring energy, is conceptually supported by Cousins et al. (2009) and Seamon (1984) psychological definitions of sublime emotion in nature. This dual emotion is connected to other spiritual and transcendent feelings, like Maslow’s peak experience (1964) and Csikszentmihalyi (2014) deep flow experience.

Sublime emotion toward nature differs conceptually and operationally from other, similar variables, like feelings of love and care for nature, emotional affinity toward nature, and connectedness with nature. For instance, inspiring energy and Perkins (2010) notion of love and care for nature could have some shared content but they refer to different feelings during wilderness experiences. In all likelihood, the content of Perkins (2010) concept is more closely aligned with emotions triggered by inspiring energy, because they share contents of identification and connectedness with nature, and good feelings. These two variables would logically, therefore, be highly correlated, suggesting potential collinearity between them, may be due to the similarity of contents in the identification, union with nature and sensation of positive elements. However, although they share certain emotional contents, the inspiring energy and the love and care for nature are not the same variable, since they respond to functions different from the experience of nature. The content of the love and care for nature (operative measure) is specific for the care and commitment of the environment, but not those of the inspiring energy, which do not make direct mention of the environmental care.

Love and care for nature also differs from awe. The only awe-related item on the Love and Care for Nature scale (item 11, *a menudo me surgen sentimientos de temor y admiración cuando estoy en la naturaleza* [I often feel a sense of awe and wonder when I am in unspoilt nature]) did not load onto its single factor and had to be eliminated from this study’s results. Feeling love and feeling awe, toward nature, are two different emotions people experience upon contact with it, or upon remembering a wilderness experience. In fact, the awe factor may be more closely tied to a person’s inner, private world (Shiota et al., 2007), whereas feeling love and care for nature develops under conditions of pleasure and appreciation for a “beautiful,” “attractive,” and “pleasurable” wilderness experience (Armstrong and Detweiler-Bedell, 2008), and the express desire to care for and protect it (Perkins, 2010).

### Sublime Emotion Toward Nature in Relation to Other Variables

Regarding the relationship between inspiring energy and awe, and environmentalism variables, we found that both of them positively and significantly predict environmental behaviors. These results offer evidence of sublime emotion toward nature’s criterion validity because awe and inspiring energy both have a significant predictive effect on environmental behaviors. That is a novel finding within the literature; to date, there are no earlier results to which to compare.

That being said, we must emphasize that the constituent factors of sublime emotion toward nature do not jointly explain environmental behaviors. Inspiring energy is probably more similar to *core affect*, proposed by Russel and Feldman (1999), because it includes emotional activation, as in joy, vitality, happiness, freedom, harmony, and energy. Awe, on the other hand, may involve greater information processing, akin to a full-blown prototypical emotional episode as described by Russel and Feldman (1999), since it includes vulnerability, humility, a feeling of veneration for the mysteries of nature – feelings that might lead to lasting personal change (Shiota et al., 2007). Therefore, we leave it to future research to test the hypothesis that inspiring energy more closely corresponds to emotional impulses triggered by wilderness experience, or memory thereof, whereas awe is a more complex emotion that, above and beyond core affect, involves a prototypical emotional episode.

Another interesting result was the positive and significant relationship between awe, environmental apathy and anthropocentrism. This result suggests that awe, environmental apathy and anthropocentrism could share an element in common, related to selfish motivations, fear as a process of personal growth that could not necessarily be directed toward the benefit of the external to the individual (Seamon, 1984; Shiota et al., 2007). However, awe is the only of these variables positively related to inspiring energy. Thus, feeling reverential fear of nature, vulnerability to its presence is related to a feeling of rejoicing, wellbeing, vitality, personal freedom and connection with nature, but not necessarily related to the desire and commitment to make personal sacrifices in favor of environmental care.

### Future Developments

We can conclude that sublime emotion toward nature is a kind of transcendence, but not all transcendent experiences are sublime feelings toward nature. By definition, sublime emotion toward nature can include a series of transcendent emotions, considering it fuses concepts equivalent to Williams and Harvey (2001) transcendent emotions, peak experience (Maslow, 1964), and flow state (Csikszentmihalyi, 2014). According to those authors, self-transcendence is characterized by intense positive affect beyond the everyday, feeling as one with the universe, and sensing a higher power or being (Williams and Harvey, 2001).

Furthermore, the two factors of sublime emotion toward nature we found in this study –awe and inspiring energy – feed the emotional bond with nature. That process might not lead only to environmentalist commitment, instead prompting additional psychological processes to unfold, such as transcendent change and self-realization (Maslow, 1964; Fredrickson and Anderson, 1999; Williams and Harvey, 2001), psychological (Nisbet et al., 2009) and spiritual wellbeing (Johnson, 2002; Ashley, 2007), psychological restoration (Kaplan, 1995; Kaplan and Kaplan, 1989, 2009), and activation of altruistic motivation (Piff et al., 2015; Salgado and Oceja, 2011).

Awe and inspiring energy suggest the possibility of the duality of sublime emotion toward nature: inspiring energy provoking externally oriented activities, and awe, conversely, prompting an inner, personal process (Shiota et al., 2007).

One limitation of this study is the possibility that the Sublime Emotion toward Nature scale would not capture co-occurrence of awe and inspiring energy. They are two different emotions, but at some point in the period of time they are felt, they could complement each other and happen simultaneously.

We recommend further research on the differential as well as joint effects of the factors of sublime emotion toward nature, and the wilderness settings that evoke the sublime. It is also important to keep unpacking the relationship between sublime emotion toward nature and environmentalism, and other positive psychological aspects like wellbeing and psychological restoration during wilderness experiences.

Finally, it is necessary more studies with a larger sample for improving the statistical power of the sublime emotion toward nature measure. In this sense, it is important to know an analysis of variance of sublime emotion toward nature between groups of age and others socio psychological variables, including cultural differences, according to the transcendence emotions literature.

## Ethics Statement

An ethics approval was not required at the time the study was conducted as per applicable institutional and national guidelines. However, this work is carried out within the framework of the studies planned in the project “Sublime experience and environment: psychological analysis of transcendent emotions in relation to nature” (PSI-2013-44939-P), approved for the evaluating committee from the Secretariat Of State Of Research, Development And Innovation of the Government of Spain, with the maximum qualification “A.” The research team undertook to guarantee the anonymity of the answers. All subjects gave informed consent about the procedure, duration and the objectives of the study. Each participant only answered an anonymous questionnaire, and they were free to decline to participate from the research at any time. The information was treated with confidential and only use for research purpose.

## Author Contributions

LB and JC contributed to all sections in the development of the research and the manuscript. LB conceived the first idea and designed the first instrument to measure the sublime emotion of nature. LB and JC contributed to the final version of the instrument.

## Conflict of Interest Statement

The authors declare that the research was conducted in the absence of any commercial or financial relationships that could be construed as a potential conflict of interest.
